# Neonatal outcomes of singleton live births with vanishing twin syndrome following double embryos transfer in assisted reproductive technology: a retrospective cohort study

**DOI:** 10.1186/s13048-019-0539-8

**Published:** 2019-07-20

**Authors:** Junfang Yan, Yichun Guan, Hongfang Fan, Mingkun Mu, Simin Sun, Wenjun Geng, Wei Zheng, Zhiying Xiao

**Affiliations:** The Third Affiliated Hospital of Zhengzhou University, Zhengzhou City, China

**Keywords:** Preterm birth, Low birthweight, Assisted reproductive technology, Single embryo transfer, Fresh embryos transfer cycles, Frozen embryos transfer cycles

## Abstract

**Background:**

Women with vanishing twin syndrome are associated with increased risks of adverse neonatal outcomes, such as preterm birth (PTB) and low birthweight (LBW), compared with those in singleton live births following single embryo transfer (SET) in assisted reproductive technology (ART).

**Methods:**

Anonymized data on all cycles performed in China were obtained from the Reproductive Medicine Department at the Third Affiliated Hospital of Zhengzhou University, which had involved 127597 cycles following double embryos transfer (DET), including 54585 fresh embryos transfer (ET) cycles and 73012 frozen embryos transfer (FET) cycles. In addition, the obstetric outcomes, such as gestation age, PTB, small for gestation age (SGA), birthweight (BW), LBW, congenital malformation, pediatric admission and Neonatal Intensive Care Unit (NICU) admission in the fresh ET and FET cycles, were analyzed. Moreover, logistic regression analysis was performed to adjust the confounders, including age of women, body weight index (BMI), value of AMH, infertile years, current cycle, antral follicles, cause of infertility, number of oocytes retrieved, endometrial thickness at the date of transplantation, number of high-quality embryos, and embryo stage.

**Results:**

In the fresh ET cycles, the BW and gestational age in study group were lower than those in control group, which were (2962.4 ± 563.1vs. 3104.9 ± 498. 5, *p* = 0.000) and (262.8 ± 8.4 vs. 268.9 ± 13.9, *p* = 0.000), respectively. Relative to control group, the study group was linked with increased risks of PTB (adjusted odds ratio (aOR) 2.45, 95% CI:1.98–3.03, adjusted *p* = 0.000), LBW (aOR2.11, 95% CI:1.67–2.65, adjusted *p* = 0.000), pediatric admission (aOR 2.55, 95% CI2.07–3.13, adjusted *p* = 0.000), and NICU admission (aOR 1.98, 95% CI1.32–2.96, adjusted *p* = 0.001), but there were no statistically significant differences in the risks of SGA (aOR 1.09, 95% CI0.82–1.45, adjusted *p* = 0.960) and congenital malformation (aOR 0.94, 95% CI0.53–1.68, adjusted *p* = 0.640) between the two groups. In the FET cycles, the gestational age and BW in study group were lower than those in control group, which were (263.0 ± 15.7vs. 273.0 ± 10.5, *p* = 0.000) and (3099 ± 662.1vs. 3352 ± 671.5), respectively. The study group was associated with increased risks of PTB (aOR2. 45, 95% CI: 2.23–3.43, adjusted *p* = 0.000), LBW (aOR 2.67, 95% CI: 2.13–3.34, adjusted *p* = 0.000), pediatric admission (aOR2.62, 95% CI2.14–3.21, adjusted *p* = 0.000), and NICU admission (aOR 2.22, 95% CI1.43, 3.46, adjusted *p* = 0.001) compared with those in control group, but differences in the risks of SGA (aOR 0.98, 95% CI0.71–1.36, adjusted *p* = 0.730) and congenital malformation (aOR 0.99, 95% CI 0.60,1.63, adjusted *p* = 0.940) between the two groups were not statistically significant.

**Conclusions:**

Our study finds that singleton live births with VTS have higher risks of LBW, PTB, pediatric admission and NICU admission than those without VTS in both the fresh and frozen cycles, even after adjusting for confounders. However, no increased risks of SGA or congenital malformation are observed in singleton live births in both the fresh and frozen ART cycles following DET.

## Background

Assisted reproductive technology (ART) is under rapid development since the birth of the first infant through the in-vitro fertilization (IVF) technique. This marked the practice from sophisticated experimental techniques to conventional medical care. However, compared with spontaneous conceptions, ART has a low pregnancy rate, the insufficient embryos and culture techniques. Therefore, clinicians frequently transfer multiple embryos to maximize the chance of pregnancy [1, 2]. Meanwhile, ART has resulted in a dramatically increased incidence of multiple pregnancies over the past 4 decades [3]. Specifically, multiple pregnancies are indicative of an increased risk of preterm birth (PTB) and perinatal death, which are recognized as the adverse outcomes related to the interventions of ART [4, 5]. Some national policies and guidelines usually recommend selective single embryo transfer (SET) for women with favorable prognosis and those aged less than 35–38 years [6, 7]. Typically, SET can markedly reduce the rate of multiple pregnancies following ART [8]. Nowadays, successful outcomes have been increasingly defined as the healthy monocyesis and singleton live births [9, 10]. Yet, we are concerned about the outcomes of single pregnancy throughout the process of pregnancy. So, what is the difference between a single pregnancy with vanishing twin syndrome (VTS) and that without VTS? In 1945, Stoeckel had first proposed the spontaneous reduction of a fetus in a twin pregnancies, which was referred to as the phenomenon of “vanishing twins.” Dickey et al. found that 50% patients who had 3 or more gestational sacs would have spontaneous reductions before the first 12 weeks of pregnancy, and these patients were linked with higher risks of PTB and LBW [11]. It is suggested in one study that, people with spontaneous reduction of the initial multiple pregnancies to a singleton pregnancy were associated with increased risks of adverse prenatal outcomes, such as PTB and LBW, compared with those of singleton live births with SET [12]. In addition, some other studies show that the risk of fetal growth restriction (FGR) with VTS in an early twin pregnancy is elevated relative to that in the initial single pregnancy, and the later occurrence of VTS was related to a higher risk [13]. Nonetheless, no existing study has specially delineated the adverse obstetric outcomes of twin pregnancy with VTS. Therefore, the current retrospective cohort study was carried out to compare the neonatal outcomes in singleton live births between groups with and without VTS following double embryos transfer (DET).

## Methods

### Populations

**Fig. 1:**
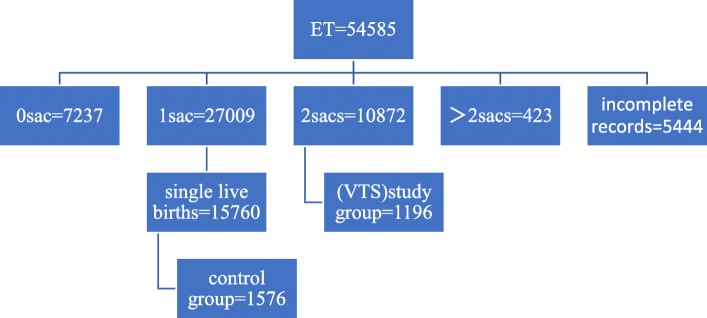
Number of included and excluded cycles in ET cycles

**Fig. 2:**
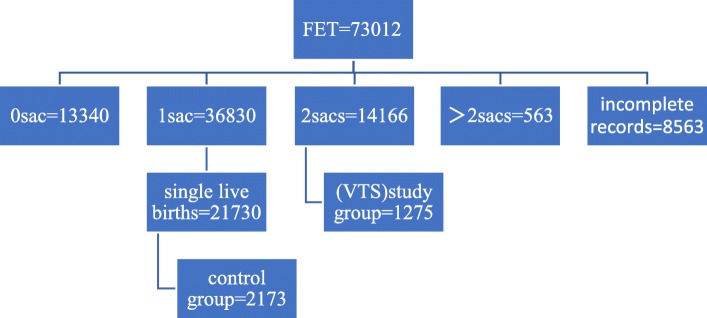
Number of included and excluded cycles in FET cycles

From January 1st, 2005 to October 1st, 2018, anonymized data on all cycles performed in China were obtained from the Reproductive Medicine Department at the Third Affiliated Hospital of Zhengzhou University, which had involved 127597 cycles following DET, including 54585 fresh ET cycles and 73012 FET cycles. Typically, the infertile couples with tubal factors or male factor (such as lean and weak sperm disease) were included in this study. Moreover, women with multiple births, uterine diseases, endocrine and medical diseases, ovarian diseases, and pre-implantation genetic diagnosis/screening (PGD/S) were excluded from this study. Furthermore, cycles with donor oocytes, donor embryos, and incomplete records were also ruled out of this study. For our study, the ART cycles with DET that resulted in singleton live births would be analyzed for their obstetric outcomes, including gestation age, PTB, SGA, BW, LBW, congenital malformation, pediatric admission and NICU admission in both fresh ET and FET cycles. Additionally, the fetal heart rate at 7 weeks of gestation was monitored with three-dimensional (3D) ultrasound, and cycles with two fetal hearts and two gestational sacs were enrolled as the study group. In addition, cycles with single fetal heart and single gestational sac were included as the control group, from which 1576 and 2173 cases were extracted at a ratio of 10:1 using the system sampling method. (Details are presented in Figs. 1 and 2). Baseline characteristics of both cohorts in fresh ET and FET cycles are presented in Table 1, including maternal age (year), body mass index (BMI), infertile year, cause of infertility (tubal disease or male factor), current cycle, blood follicle-stimulating hormone (bFSH), bE2, blood luteinizing hormone (bLH), prolactin (PRL), anti-mullerian hormone (AMH), antral follicle count (AFC), number of oocytes retrieved, number of available embryos, number of high quality embryos, stage of embryo transfer, endometrial thickness at the date of transplantation, and mode of delivery (cesarean section and vaginal delivery).

**Table 1:** Baseline characteristics of the two cohorts in fresh ET and FET cycles

	Fresh cycles				Frozen cycles			
	Study group *N* = 1196	Control group *N* = 1576	*P* value	t/x^*2*^ value	Study group *N* = 1275	Control group *N* = 2173	*P* value	t/x^*2*^ value
Age	29.5 ± 3.9	29.8 ± 4.2	0.031*	−2.16	30.7 ± 4.9	30.2 ± 4.8	0.001*	3.20
<35	1094 (91.5)	1406 (89.2)	0.061	3.47	913 (71.6)	1586 (73.0)	0.698	0.70
≥35	102 (8.5)	117 (10.8)			362 (28.4)	587 (27.0)		
BMI	22.9 ± 3.0	23.0 ± 3.1	0.15	−1.45	23.0 ± 2.5	22.8 ± 2.3	0.016*	2.42
Infertile year	1.8 ± 1.3	1.8 ± 1.8	0.86	0.18	2.5 ± 1.5	2.7 ± 1.5	0.001*	3.23
Cause of infertility			0.314	1.01			0.177	0.82
Tubal factor	788 (65.9)	1067 (67.7)			826 (64.8)	1458 (67.1)		
Male factor	408 (34.1)	509 (32.3)			449 (35.2)	715 (32.9)		
Current cycle	1.4 ± 0.7	1.4 ± 1.1	0.96	0.055	2.4 ± 1.8	2.4 ± 1.6	0.194	1.30
bFSH	7.0 ± 2.2	7.0 ± 3.7	0.84	−0.21	7.9 ± 2.6	7.7 ± 1.8	0.057	1.91
bE2	37.1 ± 8.9	36.5 ± 9.1	0.101	1.64	48.7 ± 16.0	49.5 ± 12.4	0.146	−1.45
bLH	4.6 ± 3.3	4.3 ± 2.8	0.21	1.82	4.4 ± 2.5	4.3 ± 2.1	0.201	1.28
PRL	414.7 ± 178.8	402.0 ± 204.8	0.089	1.703	4.9.3 ± 254.9	409.9 ± 184.8	0.939	−0.077
AMH	5.4 ± 0.60	5.3 ± 0.67	0.029*	0.089	5.1 ± 2.6	5.3 ± 2.8	0.068	−1.824
AFC	15.7 ± 4.4	15.5 ± 5.4	0.55	0.60	/	/	/	/
Number of oocytes retrieved	10.5 ± 4.5	10.6 ± 3.9	0.53	0.62	/	/	/	/
Number of available embryos	8.2 ± 1.2	8.0 ± 1.6	0.01*	2.57	/	/	/	/
Number of high-quality embryos	5.1 ± 2.6	4.8 ± 2.4	0.003*	2.95	/	/	/	/
embryo stage			0.000*	126.4			0.01*	6.57
cleavage-stage embryo	1015 (84.9)	1040 (66.0)			874 (68.5)	1395 (64.2)		
blastocyst	181 (15.1)	536 (34.0)			401 (31.4)	778 (35.8)		
endometrial thickness at the day of transplantation	11.6 ± 1.3	11.7 ± 1.1	0.002*	−3.1	10.3 ± 1.9	10.2 ± 1.5	0.091	1.69
mode of delivery			0.000*	67.0			0.000*	210.9
cesarean section	766 (64.0)	1233 (78.2)			851 (66.7)	1899 (87.4)		
vaginal delivery	430 (36.0)	343 (21.8)			424 (33.3)	274 (12.6)		

*p < 0.05 was statistically significant

**Table 2:** Neonatal outcomes of singleton live births in fresh ET and FET cycles

Fresh cycles							Frozen cycles					
	Study group N = 1196	Control group N = 1576	*P* value	OR, 95% CI	Adjusted *P* value	Adjusted OR, 95% CI	Study group N = 1275	Control group N = 2173	*P* value	OR, 95% CI	Adjusted *P* value	Adjusted OR, 95% CI
Gestational age	262.8 ± 8.4	268.9 ± 13.9	0.000	/	/	/	263.0 ± 15.7	273.0 ± 10.5	0.000	/	/	/
PTB	258 (21.6)	159 (10.1)	0.000	2.45(1.98–3.03)	0.000*	2.41(1.93–2.99)	230 (18.0)	160 (7.4)	0.000	2.77 (2.23,3.43)	0.000*	2.68 (2.15,3.33)
birthweight	2962.4 ± 563.1	3104.9 ± 498.5	0.000	/	/	/	3099 ± 662.1	3352 ± 671.5	0.000	/	/	/
LBW	201(16.8)	138 (8.8)	0.000	2.11 (1.67–2.65)	0.000*	2.21 (1.74–2.80)	204 (16.0)	145 (6.6)	0.000	2.67 (2.13–3.34)	0.000*	2.07 (2.12,3.35)
SGA	90 (7.5)	111 (7.1)	0.561	1.09 (0.82–1.45)	0.960	1.01 (0.75,1.35)	60 (4.7)	104 (4.8)	0.915	0.98 (0.71–1.36)	0.730	0.94 (0.68,1.31)
Congenital malformation	20 (1.7)	28 (1.8)	0.835	0.94 (0.53–1.68)	0.640	0.87 (0.48–1.57)	25 (2.0)	43 (19.8)	0.971	0.99 (0.60,1.63)	0.940	0.98 (0.59,1.62)
Pediatrics admission	280 (23.4)	169 (10.7)	0.000	2.55 (2.07–3.13)	0.000*	2.62 (2.12–3.24)	256 (20.1)	190 (8.7)	0.000	2.62 (2.14–3.21)	0.000*	2.60 (2.12,3.19)
NICU admission	60 (5.0)	26 (1.6)	0.001	1.98 (1.32,2.96)	0.001*	2.04 (1.36,3.08)	46 (3.6)	36 (1.7)	0.000	2.22 (1.43,3.46)	0.001*	2.21 (1.41,3.44)

*p < 0.05 was statistically significant. Adjusted for confounders, including age of women, BMI, value of AMH, infertile years, current cycle, AFC, cause of infertility, number of oocytes retrieved, endometrial thickness at the day of transplantation, number of high-quality embryos, embryo stage

### Embryo transfer and follow-up

2 fresh or frozen embryos would be transplanted into the uterus of each patient by doctors. Subsequently, from that day, the patient receives luteal support treatment,including progesterone injection and vaginal progesterone release gel, and gradually reduced after 45 days of transplantation. Beta human chorionic gonadotropin (β-hCG) in blood was tested at 14 days after DET. Professionals at the hospital would obtain patient information by means of telephone interview and retrieval of the hospitalization system throughout the process from pregnancy to birth; afterwards, they would upload all materials into the Reproductive Center Database.

The primary endpoints of our study were obstetric outcomes, including PTB (gestational age of<37 weeks), small for gestation age (SGA), LBW (BW of<2500 g), congenital malformation (such as Trisomy 13/18/21, congenital heart disease (CHD), polydactyly/syndactyly and others), pediatric admission (transferred to pediatrics after birth), and neonatal intensive care unit (NICU) admission ((transferred to NICU after birth).

### Statistical analysis

Data were analyzed using the SPSS 21.0 statistical software. The cohort characteristics were described using the chi-square test for categorical variables, while continuous variables were expressed as means ±SD. *P* < 0.05 was statistically significant. Logistic regression analysis was performed to adjust the confounders, including maternal age, BMI, value of AMH, infertile years, current cycle, AFC, cause of infertility, number of oocytes retrieved, endometrial thickness at the date of transplantation, number of high-quality embryos, and embryo stage.

## Results

### Baseline characteristics

Baseline characteristics of the cohorts in both fresh and frozen cycles are illustrated in Table 1.

Firstly, for the fresh ET cycles, the maternal age in control group was higher than that in study group (29.8 ± 4.2 VS 29.5 ± 3.9, *p* = 0.031). At the same time, the value of AMH was also higher in study group compared with that in control group (5.4 ± 0.60 VS 5.3 ± 0.67, *p* = 0.029). Moreover, more high quality embryos obtained from the fresh cycles and more cleavage-stage embryos were included in study group than those in control group, which were (5.1 ± 2.6 VS 4.8 ± 2.4, *p* = 0.003) and (84.9% V S66.0%, *p* = 0.000), respectively. But the endometrial thickness at the date of transplantation in control group was greater than that in study group (11.7 ± 1.1 VS 11.6 ± 1.3, *p* = 0.002). More women received cesarean section in control group than in study group (78.2 VS 64%, *p* = 0.000) (Table 1).

Secondly, for the FET cycles, the maternal age and BMI were higher in study group than in control group, which were (30.7 ± 4.9 VS 30.2 ± 4.8, *p* = 0.001) and (23.0 ± 2.5 VS 22.8 ± 2.3, *p* = 0.0016), respectively. In addition, the infertile year was lower in study group compared with that in control group (2.5 ± 1.5 VS 2.7 ± 1.5, *p* = 0.001). Besides, there were more cleavage-stage embryos included in study group than those in control group (68.5% VS 64.2%, *p* = 0.01), and more infants are delivered through cesarean section in control group than in study group (87.4% VS 66.7%, *p* = 0.000) (Table 1).

### Primary outcomes

#### Neonatal outcomes of singleton live births in the fresh ET cycles

In the fresh ET cycles, the BW and gestational age in study group were lower than those in control group, which were (2962.4 ± 563.1 vs. 3104.9 ± 498.5, *p* = 0.000) and (262.8 ± 8.4 vs. 268.9 ± 13.9, *p* = 0.000), respectively. Relative to control group, the study group was linked with increased risks of PTB (adjusted odds ratio (aOR) 2.45, 95% CI:1.98–3.03, adjusted *p* = 0.000), LBW (aOR2.11, 95% CI:1.67–2.65, adjusted *p* = 0.000),pediatric admission (aOR 2.55, 95% CI2.07–3.13, adjusted *p* = 0.000), and NICU admission (aOR 1.98,95% CI1.32–2.96, adjusted *p* = 0.001), but there were no statistically significant differences in the risks of SGA (aOR 1.09,95% CI0.82–1.45, adjusted *p* = 0.960) and congenital malformation (aOR 0.94, 95% CI0.53–1.68, adjusted *p* = 0.640) between the two groups (Table 2).

#### Neonatal outcomes of singleton live births in the FET cycles

In the FET cycles, the gestational age and BW in study group were lower than those in control group, which were (263.0 ± 15.7 vs. 273.0 ± 10.5, *p* = 0.000) and (3099 ± 662.1 vs. 3352 ± 671.5), respectively. The study group was associated with increased risks of PTB (aOR2.45, 95% CI: 2.23–3.43, adjusted *p* = 0.000), LBW (aOR 2.67, 95% CI: 2.13–3.34, adjusted *p* = 0.000), pediatric admission (aOR2.62, 95% CI2.14–3.21, adjusted *p* = 0.000), and NICU admission (aOR 2.22, 95% CI1.43,3.46, adjusted *p* = 0.001) compared with those in control group, but differences in the risks of SGA (aOR 0.98,95% CI0.71–1.36, adjusted *p* = 0.730) and congenital malformation (aOR 0.99, 95% CI0.60, 1.63, adjusted *p* = 0.940) were not statistically significant between the two groups (Table 2).

## Discussion

It is discovered in this retrospective cohort study that, for both fresh and frozen cycles, the study groups have increased risks of LBW, PTB, pediatric admission and NICU admission relative to those in control groups. However, no increased risks of SGA or congenital malformation are observed in singleton live births in both the fresh and frozen ART cycles following DET.

This study has provided an important supplement to the existing literature, which confirms that VTS may result in higher risks of LBW, PTB, pediatric admission and NICU admission. Our findings are consistent with the conclusions obtained from previous studies, systematic reviews and meta-analyses. However, no study has recruited infertile couples with only tubal factors or male factor (lean and weak sperm disease). Such a study design is critical and necessary, which may avoid uncontrolled bias. For instance, polycystic ovary syndrome (PCOS) has been recognized as an endocrine disease related to the increased risk of adverse perinatal outcomes [14, 15]. Sharma et al. found that the presence of adenomyosis might contribute to the adverse effects on the IVF outcomes in terms of the clinical pregnancy rate, live birth rate and miscarriage rate [16]. Additionally, Wang LF et al. suggested that pre-pregnancy obesity might result in the high prevalence of macrosomia, which increased the mean BW in cohort analysis [17]. Therefore, it is crucial to recruit the infertile patients with tubal factors and male factors alone, to investigate the differences between the two groups in our study. Also, this study has particularly compared the perinatal outcomes in singleton live births between the groups with initial single sac and initial double sacs following DET. Our results has indicated higher risks of LBW and PTB between patients with and without VTS following DET in both the fresh and frozen cycles. According to an earlier study, compared with single births obtained from SET, PTB and LBT infants are more likely to develop spontaneous reduction in the initial multiple pregnancies to singleton fetuses following transfer of multiple embryos [12]. Almoq. B et al. held that pregnancy with VTS was linked with adverse obstetric outcomes (PTB and LBW) relative to those in the initial singleton pregnancy of IVF [18]. Furthermore, SUN et al. believed that the VTS survivors displayed a higher incidence of LBW than that in singleton fetuses from single pregnancies in IVF-ET [19]. Besides, it is also suggested in previous results that, the early death of a pair of twins may improve the pregnancy outcomes. But there may be more adverse complications when continuing the twin pregnancy [20]. Analysis by Timur H et al. indicated that VTS patients were more likely to develop LBW, very low birthweight (VLBW), intrauterine growth restriction and pre-eclampsia [21–23]. What’s more, Zhu Y et al. revealed that VTS could affect the obstetric outcomes in survivors, but the impact of VTS was unstable. Yet it is too early to conclude that VTS will produce adverse obstetric outcomes, such a statement may also decrease their anxiety with VTS [24]. Our study shows that the rate of cesarean section is higher in the control group, because of the strong demand of women for cesarean section. Treatment of infertile couples with years of ART has brought about great panic, and these couples are more willing to choose cesarean section instead of facing the uncertainty of delivery, which is quiet common in China. Doctors will also agree with them. Fetal weight is also estimated before delivery, and there are many macrosomia and infants of normal BW, so most women are more willing to choose cesarean section to avoid the risk of huge children .

In this study, there are no statistically significant differences in SGA and congenital malformation between the two groups in both fresh and frozen ART cycles following DET, which may be affected by the strict inclusion criteria. Probably, such results may be caused by the fact that people choose to induce labor when SGA (especially in the case of abnormal chromosome) and congenital malformation are detected in middle pregnancy. Conversely, Luke suggested that the risk of moderate growth restriction in singletons was increased following transfer of multiple embryos relative to that after SET, thus demonstrating a significant adverse effect on the intrauterine growth following the multiple embryos transfer. However, it remains unclear about whether such effect can be ascribed to the compromised embryo quality, degenerated implantation sites, or other factors [25]. Typically, the higher incidence of VTS may account for the possible mechanism affecting the incidence of LBW in the study group. La Sala et al. suggested that VTS induced a deleterious effect on the ongoing pregnancy due to blood shunting from vascular anastomoses in the surviving twin placenta [26]. Besides, we found that after the dead embryo at middle or late pregnancy came out of the uterus, the surviving embryo were born in a short time, which might increase the risk of PTB, reduce the average BW, and elevate the possibilities of pediatrics admission as well as NICU admission. Nonetheless, such event is extremely rare (about 1/1000), which does not make an increase the risk of adverse outcomes. Most VTS occur in early pregnancy (7 weeks to 12 weeks), which is the real dominant factor of adverse outcomes. Additionally, chronic inflammation may account for the other speculation for the impact of VTS on the surviving twin [27]. Regrettably, no explanation is available for such speculation.

### Strengths and limitations

The strengths of our research are as follows: all cycles are performed in the single center, which might reduce the internal bias (e.g., the laboratory technology, and operational procedures). Furthermore, we targets cycles with the tubal and male factors and rule out cycles with other infertility factors. However, previous relevant studies comparing neonatal outcomes between these groups did not exclude these cycles.

Some limitations exist in this study. One is the inherent characteristic of retrospective cohort study. Moreover, we do not collect information about the potential confounders, such as maternal smoking, drinking, previous history of abortion and different medical conditions during pregnancy. Additionally, further studies need to be designed to explore potential biological mechanism associated with adverse obstetric outcomes.

## Conclusions

Our study finds that singleton live births with VTS have higher risks of LBW, PTB, pediatric admission and NICU admission than those without VTS in both the fresh and frozen cycles, even after adjusting for confounders. However, no increased risks of SGA or congenital malformation are observed in singleton live births in both the fresh and frozen ART cycles following DET.

## Data Availability

All data were included in this article.

## Abbreviations

ART: Assisted Reproductive Technology
FET: Frozen embryo transfer
LBW: Low birthweight
PTB: Preterm birth
SET: Selective embryo transfer
VTS: Vanishing twin syndrome
